# Stress-induced aberrations in sensory processing predict worse cognitive outcomes in healthy aging adults

**DOI:** 10.18632/aging.203433

**Published:** 2021-08-18

**Authors:** Rachel K. Spooner, Brittany K. Taylor, Emma L’Heureux, Mikki Schantell, Yasra Arif, Pamela E. May, Brenda Morsey, Tina Wang, Trey Ideker, Howard S. Fox, Tony W. Wilson

**Affiliations:** 1 Institute for Human Neuroscience, Boys Town National Research Hospital, Omaha, NE 68010, USA; 2 College of Medicine, University of Nebraska Medical Center, Omaha, NE 68198, USA; 3 Department of Neurological Sciences, University of Nebraska Medical Center, Omaha, NE 68198, USA; 4 Department of Medicine, University of California San Diego, La Jolla, CA 92161, USA

**Keywords:** somatosensory, magnetoencephalography, neuropsychological assessment, allostatic load, DNA methylation

## Abstract

It is well recognized that not all individuals age equivalently, with functional dependence attributable, at least in part, to stress accumulated across the lifespan. Amongst these dependencies are age-related declines in cognitive function, which may be the result of impaired inhibitory processing (e.g., sensory gating). Herein, we examined the unique roles of life and biological stress on somatosensory gating dynamics in 74 adults (22-72 years old). Participants completed a sensory gating paired-pulse electrical stimulation paradigm of the right median nerve during magnetoencephalography (MEG) and data were subjected to advanced oscillatory and time-domain analysis methods. We observed separable mechanisms by which increasing levels of life and biological stress predicted higher oscillatory gating ratios, indicative of age-related impairments in inhibitory function. Specifically, elevations in life stress significantly modulated the neural response to the first stimulation in the pair, while elevations in biological stress significantly modulated the neural response to the second stimulation in the pair. In contrast, neither elevations in life nor biological stress significantly predicted the gating of time-domain neural activity in the somatosensory cortex. Finally, our study is the first to link stress-induced decline in sensory gating to cognitive dysfunction, suggesting that gating paradigms may hold promise for detecting discrepant functional trajectories in age-related pathologies in the future.

## INTRODUCTION

The healthy aging process is associated with a host of neurobiological changes to brain structure and function, often leading to instances of cognitive and behavioral decline and further, functional dependence in later life. Amongst these changes are age-related aberrations in local inhibitory processing (e.g., sensory gating). Briefly, sensory gating is a neurophysiological phenomenon whereby the brain’s response to a second redundant input is attenuated compared to its response to the first presentation of the stimuli [1–4]. This attenuation is thought to reflect the brain’s capacity to filter irrelevant or redundant information to preserve neural resources for more behaviorally-relevant stimuli [5]. While disturbances in sensory gating have been demonstrated in numerous clinical populations (e.g., schizophrenia, bipolar disorder, cerebral palsy) and across sensory modalities (e.g., somatosensory, auditory) [6–9], recent evidence suggests that healthy older adults and those demonstrating age acceleration (e.g., HIV-infection) exhibit impaired gating of sensory input in both primary somatosensory and higher order prefrontal regions [10–13]. Nevertheless, these deficits in bottom-up filtering mechanisms are often reflective of impaired inhibitory function and importantly, may also be predictive of changes in higher order cognitive abilities [14, 15]. Thus, it is possible that age-related cognitive decline may be attributable, at least in part, to deficits in pre-attentive inhibition. Unfortunately, the key factors driving age-related inhibitory decline remain poorly understood, highlighting the need to identify markers that more precisely characterize the neural and behavioral aberrancies in aging adults.

Healthy aging (i.e., maintenance of physiological homeostasis and stability) requires an organism to effectively adapt to changing environmental and/or biological demands (i.e., stress). For example, psychosocial and physical life stress (e.g., experience, education, income, health status) in humans is thought to be the most common precursor to physiological imbalances in the system (e.g., hormone secretion, metabolic dysfunction), which emerge in aging and are often termed allostatic load [16]. In other words, cumulative exposure to life stressors activate physiological responses, and when overloaded can lead to functional decline during the aging process. Importantly, the life stress precursors (i.e., allostatic load) that accompany this physiological dysregulation have been shown to be tightly linked to cognitive and behavioral function in aging adults [17–19]. For example, in the MacArthur studies of successful aging, older adults exhibiting greater allostatic load (defined as physiological biomarkers reflecting more stress) had worse baseline cognitive (e.g., abstraction, memory) and physical (e.g., balance) performance, as well as greater functional decline during a 3-year longitudinal follow-up than those identified with lower allostatic load [18, 20–22]. Similarly, greater allostatic load has also been associated with changes in the brain, including distributed grey and white matter volume loss in primary sensory and association cortices. However, few studies have demonstrated a direct link between these stress-related neural aberrations and the cognitive decline associated with allostasis in aging populations [17, 23, 24]. Nonetheless, these studies suggest that quantitative assessments of allostatic load (i.e., induced by life stressors) may be critical to understanding the variability in age-related alterations seen in neural and behavioral performance.

In addition to life stress, biological origins of stress may also prove informative for evaluating the variability in age-related neural and cognitive decline. One such marker that has gained popularity in recent years is the methylation of cytosine-phosphate-guanine (CpG) sites in the DNA of human tissues [25, 26]. Not only are epigenetic markers of DNA methylation highly correspondent with an individual’s chronological age (i.e., calendar time since birth) [25, 27, 28], they are also predictive of all-cause mortality rates [29], physical and cognitive ability in older age [30], as well as the pathological age acceleration (i.e., relative age acceleration) observed in neuro-degenerative diseases such as Alzheimer’s disease [31], Parkinson’s disease [32], and HIV-infection [33–35]. Together, these data suggest that epigenetic changes accumulate over the lifespan (i.e., biological stress) and may be particularly sensitive to detecting disparate aging trajectories.

**Figure 1:**
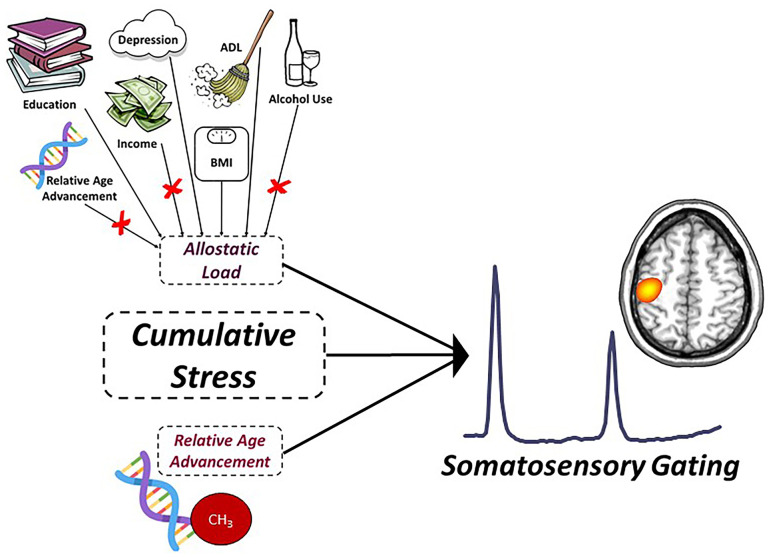
**Predictive model of sensory gating.** Conceptual figure denoting the statistical model probed in the current study. Allostatic load (i.e., life stress), relative age acceleration (i.e., biological stress) and their interaction (i.e., cumulative stress) were used to predict the age-related decline in somatosensory gating (response power to stim #2/response power to stim #1). The factors contributing to allostatic load were derived from an exploratory factor analysis (EFA) and included depression symptom severity, years of education, self-reported declines in activities of daily living, and BMI. Variables denoted with a red ‘X’ reflect measures that loaded poorly and were not included in the final factor definition for allostatic load. Relative age acceleration was quantified using the residuals from the regression of the “Consensus Model” predicted biological age on chronological age in our sample.

While age-related decline in functional inhibition (i.e., sensory gating) has been well-documented in recent years, there remain many open questions regarding the mechanisms underlying this deficit, as well as its relationship to higher order cognitive processing and the global functional decline observed across the lifespan. Thus, the goal of the current study was to determine the independent and cumulative impact of life and biological stress (i.e., allostatic load and DNA-methylation based age acceleration, respectively; Figure 1) on the neural oscillatory dynamics serving somatosensory gating (i.e., local inhibition) and cognitive function in 74 healthy adults. Specifically, adults aged 22-72 years old completed a paired-pulse electrical stimulation paradigm during magnetoencephalographic (MEG) imaging, and we applied advanced signal processing methods and structural equation modeling for hypothesis testing. Despite the most common approach for characterizing sensory gating being the evaluation of event-related potentials, several recent studies have found rich, multi-spectral neural oscillatory activity up to 90 Hz using advanced analysis approaches [6, 11, 13, 15, 36–39]. Interestingly, high frequency gamma oscillations (>30 Hz) like those elicited during somatosensory gating paradigms may rely critically on GABAergic inhibitory interneuronal pools, and thus alterations in gating of high-frequency oscillations may be directly related to changes in intracortical inhibition, making the evaluation of neural oscillations an attractive endeavor for probing functional inhibitory processing in healthy aging [40–47]. Thus, to facilitate comparison with previous studies of evoked *and* oscillatory somatosensory processing, we employed both time- and time-frequency domain approaches in this study. Our primary hypotheses were that greater levels of life (i.e., allostatic load) and biological (i.e., relative age acceleration) stress would (i) predict worse sensory gating in healthy adults, (ii) have dissociable mechanisms of action on sensory gating processes in the brain, and (iii) would more strongly affect gamma-mediated mechanisms of sensory gating compared to evoked neural responses to somatosensory stimulation.

## RESULTS

### Neural responses to somatosensory stimulation

**Figure 2:**
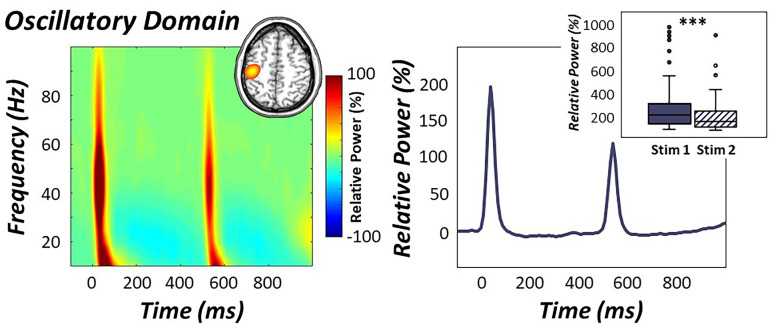
**Oscillatory response to electrical stimulation.** (Left): Time-frequency spectrogram over the sensorimotor cortices (M0443) revealed robust broadband gamma responses (i.e., 30-75 Hz) following the first and second stimulation of the right median nerve. Time is denoted on the x-axis (in ms) and frequency is denoted on the y-axis (in Hz), with units being the percent power change from baseline (-700 to -300 ms). A color scale bar denoting the directionality of this change is shown to the right of the graphic. Grand-averaged beamformer images (i.e., across all participants and both stimulations) revealed strong increases in gamma activity in the contralateral hand region of the primary somatosensory cortex (inset in top right). (Right): The neural time course of the relative power envelope (30-75 Hz band) was extracted from the peak voxel in the contralateral somatosensory cortex and is shown averaged across all participants. Oscillatory responses to the second stimulation in the pair were strongly attenuated compared to the first (box plot inset in top right), indicative of significant gating of gamma activity across all participants. ***p < .001.

Statistical analysis of our MEG sensor-level oscillatory data revealed robust broadband synchronizations in sensors near the sensorimotor cortices from about 10 to 90 Hz during the first 50 ms following onset of the first stimulation, with responses to the second stimulation (500-550 ms) extending from roughly 10 to 75 Hz (*p* < .001, corrected; Figure 2). To evaluate the dynamics, we focused our beamformer image reconstruction analysis on the higher 30-75 Hz frequency range and two 50 ms time intervals in which the oscillatory response was the strongest following stimulation (i.e., 0-50 and 500-550 ms for stimulations 1 and 2, respectively). Of note, we limited our analysis to 30 Hz on the low end because this aligns with the traditional definition of the gamma band. In contrast, we restricted our analyses to 75 Hz on the high end as the relative power sharply decreased thereafter, especially in response to the second stimulus.

Beamformer images revealed robust increases in gamma activity in the contralateral hand region of the primary somatosensory cortex (Figure 2), with identical peak locations in response to the first and second stimulation. As described in the methods, these images were grand-averaged across all participants and both stimulations to extract virtual sensor time series from the peak voxel. We derived the relative power envelope for the 30-75 Hz band from the resulting baseline-corrected time series, and used these in subsequent multilevel models of life and biological stress on gamma activity. Importantly, paired-sample t-tests between stimulation response power revealed that the response to the second stimulation was significantly reduced compared to the first, indicative of significant gating of gamma oscillatory power in the primary somatosensory cortex across all participants (*t*(73) = 6.54, *p* < .001; Figure 2).

**Figure 3:**
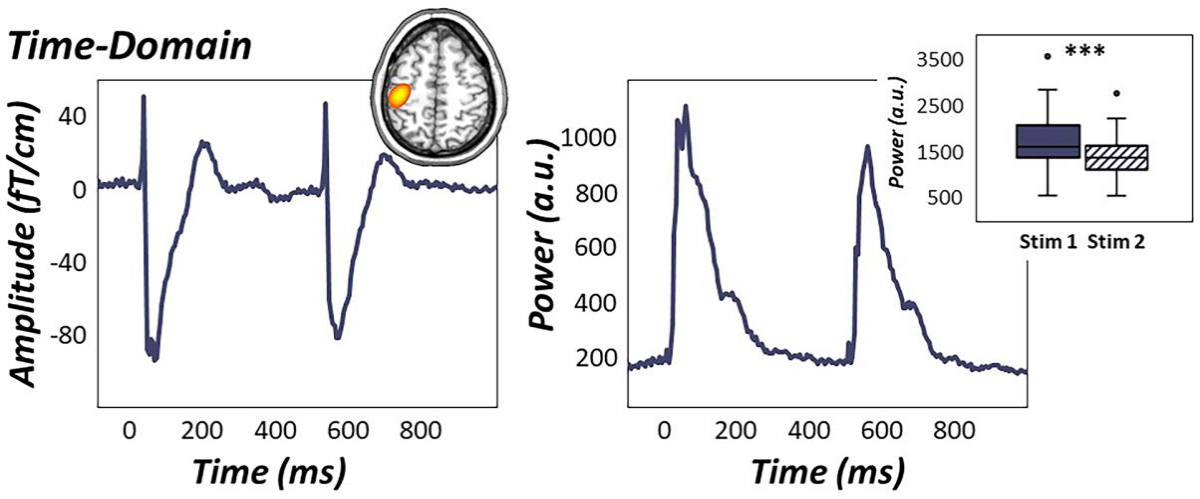
**Time-domain response to electrical stimulation.** (Left): Time domain average of data from a representative sensor near the left sensorimotor cortex (M0443). Grand-averaged sLORETA source estimates (inset in top right) of statistically-derived sensor-level temporal clusters showed robust increases in phase-locked neural activity from 25-125 ms and 525-625 ms in the left primary somatosensory cortex. (Right): The peak voxel time course revealed significantly attenuated time-domain responses to the second stimulation compared to the first, indicative of significant gating of time-domain activity across all participants during paired-pulse stimulation (Right: box plot). ***p < .001.

In contrast, evaluation of sensor-level time-domain responses revealed more temporally-extended clusters evolving shortly after each somatosensory stimulation (i.e., 25-125 ms and 525-625 ms for stimulations 1 and 2, respectively) in sensors near the contralateral sensorimotor strip (*p* < .001, corrected; Figure 3). sLORETA source images revealed robust increases in phase-locked, time-domain neural responses in the left primary somatosensory cortex contralateral to stimulation, with identical peak locations in response to the first and second stimulation. As described in the methods, these images were grand-averaged across all participants and both stimulations to extract time series from the peak voxel for subsequent multilevel models of life and biological stress on evoked data (Figure 3). Paired-sample t-tests of stimulation response power revealed significantly weaker time-domain responses to the second stimulus compared to the first, indicative of significant sensory gating in the time-domain across all participants (*t*(73) = 9.31, *p* < .001, Figure 3).

### Oscillatory profiles of somatosensory gating are modulated by life and biological stress

**Figure 4:**
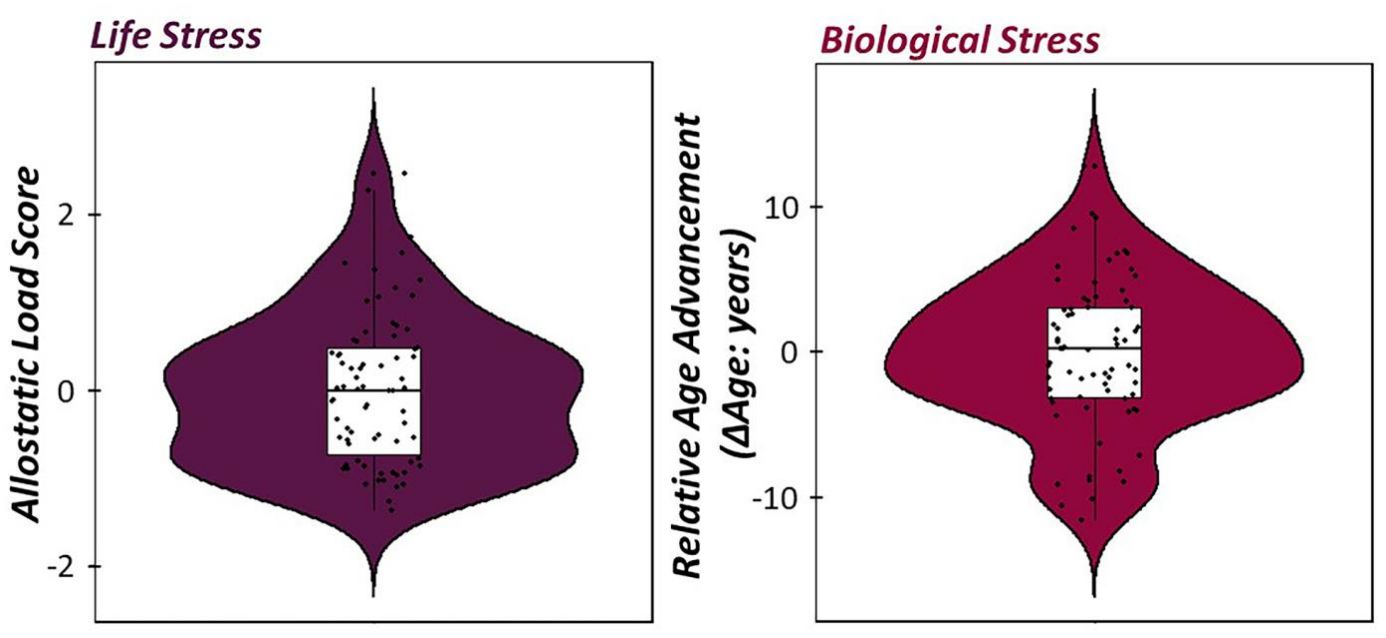
**Life and biological stress in healthy aging adults.** (Left) Violin/box plots of allostatic load score (Mean ± SD: 2.7 E -5 ± 0.85) extracted from the exploratory factor analysis of depression symptom severity, BMI, perceived declines in ADL, and total years of education with positive values indicative of elevated levels of life stress in our sample. (Right) Violin/box plots of relative age advancement (Mean ± SD: -0.18 ± 5.08), which was computed using the residuals from a regression of the consensus DNA methylation model of predicted biological age on chronological age in our sample.

**Figure 5:**
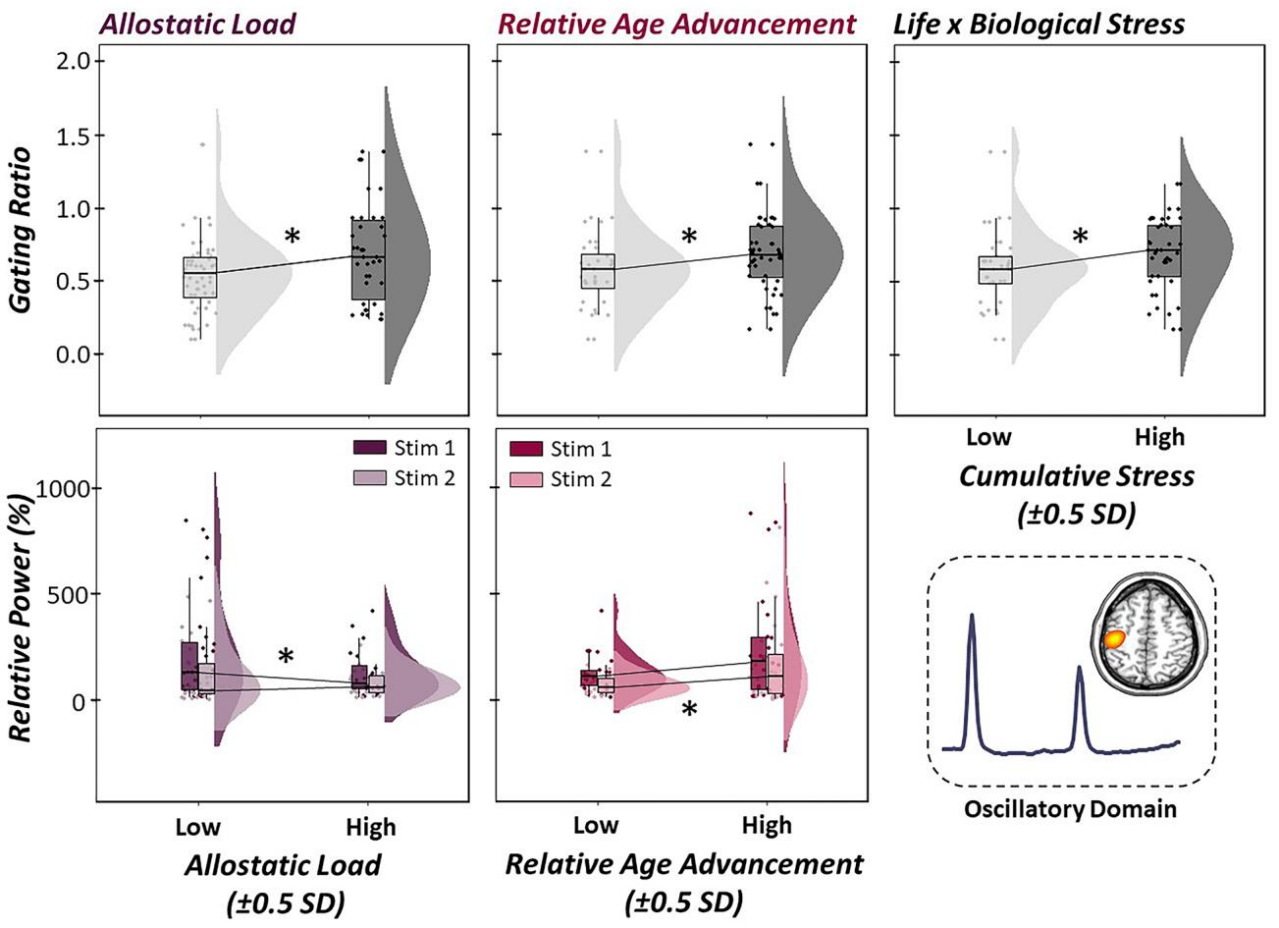
**Life and biological stress differentially predict gamma oscillatory responses.** (Top Panel): Multiple regressions of life stress (i.e., allostatic load, left), biological stress (i.e., relative age acceleration, middle) and their interaction (i.e., cumulative stress, right) on the gating of gamma activity in the primary somatosensory cortex were conducted. Raincloud plots (combined box plot, histogram distribution and individual scattered data points) denote somatosensory outcome metrics at low and high levels of each stressor (i.e., ±0.5 SDs). Life, biological and cumulative stress were all significant predictors of gating ratios, such that increased stress was associated with higher gating ratios, indicative of worse suppression of redundant sensory input. (Bottom Panel): Follow-up regressions of life and biological stress on neural response to stimulation 1 (darker color) and 2 (lighter color) in the paired-pulse paradigm revealed differential modulation of stimulation response based on stressor type. Allostatic load was significantly predictive of oscillatory responses to the first stimulation, such that higher levels of life stress led to reduced neural response to the first stimulation in the pair. In contrast, relative age acceleration was predictive of the oscillatory response to the second stimulation, such that greater biological age led to less attenuated response power to the second stimulation. All axes are fixed for each graph per row. *p < .05.

To investigate how indices of life and biological stress impacted sensory gating across the lifespan, a multiple regression of empirically-derived and sample-specific definitions of allostatic load, relative age acceleration/deceleration, and their interaction was computed on the somatosensory gating ratio derived from the gamma band relative time series. Of note, higher gating ratios are indicative of worse suppression of redundant information. For descriptive statistics regarding our empirically-derived, sample-specific measures of life and biological stress, see Figure 4. Interestingly, allostatic load, relative age acceleration/deceleration, and their interaction (i.e., cumulative stress) were significantly predictive of the integrity of sensory gating, such that increases in life, biological, and cumulative stress were predictive of higher gating ratios (i.e., worse suppression; Figure 5; allostatic load: b = 0.51, *p* = .012, 95% CI [0.18, 0.85], relative age acceleration: b = 0.43, *p* = .027, 95% CI [0.11, 0.75], cumulative stress: b = -0.02, *p* = .030, 95% CI [-0.04, -0.01].

Finally, to evaluate the mechanism of action by which life and biological stressors impact filtering in the primary somatosensory cortex, we performed structural equation modelling with allostatic load and relative age acceleration predicting gamma response power in response to the first and second stimulation, sequentially. As expected, response power to the first stimulation was significantly predictive of gamma activity in response to the second stimulation, such that increases in gamma power during the first stimulation were predictive of increased gamma power during the second stimulus (b = .60, p < .001, 95% CI [0.55, 0.65]). In regard to our measures of life and biological stress, allostatic load was significantly predictive of gamma power during the first stimulation, but not the second, such that greater levels of life stress were predictive of reduced neural response to the first stimulation in the pair (b = -.56, *p* = .043, 95% CI [-1.02, -0.11]). In contrast, relative age acceleration was significantly predictive of the neural response to the second stimulus in the pair, but not the first, such that greater age acceleration indices were predictive of greater gamma power during the second stimulation (i.e., less gating-related attenuation; b = 0.11, *p* = .014, 95% CI [0.01, 0.05]; Figure 5).

### Evoked neural response is unaffected by life and biological stress

**Figure 6:**
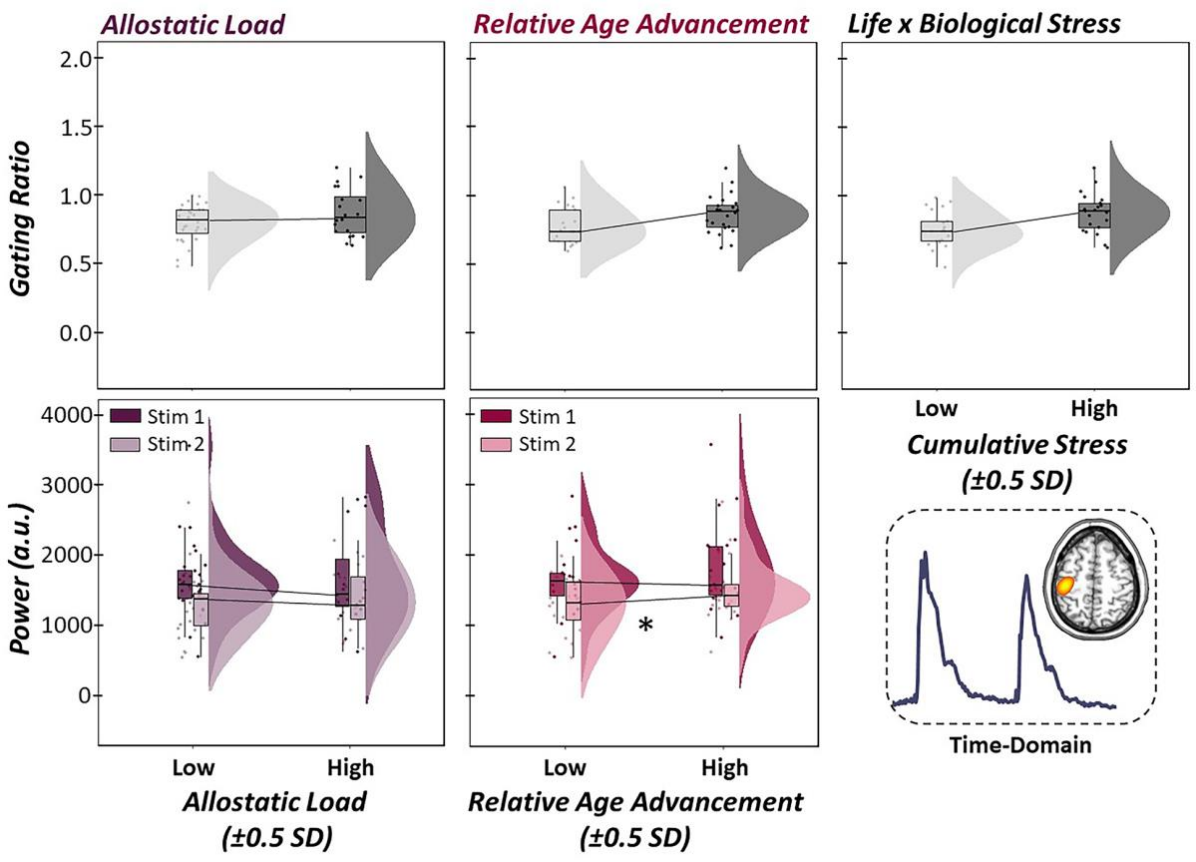
**Evoked somatosensory neural responses are unaffected by stressors.** (Top Panel): Multiple regressions of life stress (i.e., allostatic load, left), biological stress (i.e., relative age acceleration, middle) and their interaction (i.e., cumulative stress, right) on sensory gating in the time-domain. Raincloud plots (combined box plot, histogram distribution and individual scattered data points) denote somatosensory outcome metrics at low and high levels of the stressor (i.e., ±0.5 SDs). Life, biological and cumulative stress were not significantly predictive of gating in the time-domain. (Bottom Panel): Follow-up regressions of life and biological stress on neural response to stimulation 1 and 2 sequentially in the paired-pulse paradigm also showed no change in time-domain response power as a function of life stress, although increases in relative age acceleration were significantly predictive of greater neural responses to the second stimulation, but not the first. All axes are fixed for each graph per row. *p < .05.

For correspondence with the previous literature, which has focused on evoked somatosensory processing, we computed the time-domain sLORETA source images for each participant. Time series data were then extracted from the grand-averaged peak voxel of the phase-locked response estimates to derive indices of somatosensory processing (i.e., gating ratio, source power). Next, we conducted multiple regressions to evaluate whether life, biological, and cumulative stress (i.e, their interaction) predicted changes in the gating of phase-locked neural responses. Interestingly, allostatic load (b = -0.001, *p* = .993, 95% CI [-0.15, 0.15]), relative age acceleration (b = -0.001, *p* = .987, 95% CI [-0.14, 0.14]), and their interaction (b = 0.000, *p* = .913, 95% CI [-0.01, 0.01]) did not significantly predict the gating ratio derived from our time-domain analysis, suggesting that the evoked neural responses to somatosensory stimulation were not sensitive to changes in life and biological stress, as defined in the current study (Figure 6).

For completeness, we conducted the follow-up structural equation model interrogating the role of life and biological stress on the time-domain response to the first and second stimulation response power, sequentially. Similar to our oscillatory analysis, increased evoked response power to the first stimulation was significantly predictive of increased neural response power to the second stimulation (b = 0.62, *p* < .001, 95% CI [0.54, 0.69]). Interestingly, while allostatic load was not significantly predictive of phase-locked responses to the first or second stimulation (*ps* > .158), relative age acceleration was significantly predictive of neural response to the second stimulus, but not the first, such that greater age acceleration (i.e., greater biological age relative to chronological age) led to stronger neural responses to redundant stimuli (i.e., less attenuation; b = 12.91, *p* = .011, 95% CI [4.58, 21.24]; Figure 6).

### Stress-induced changes in sensory gating predict poorer cognitive outcomes

**Figure 7:**
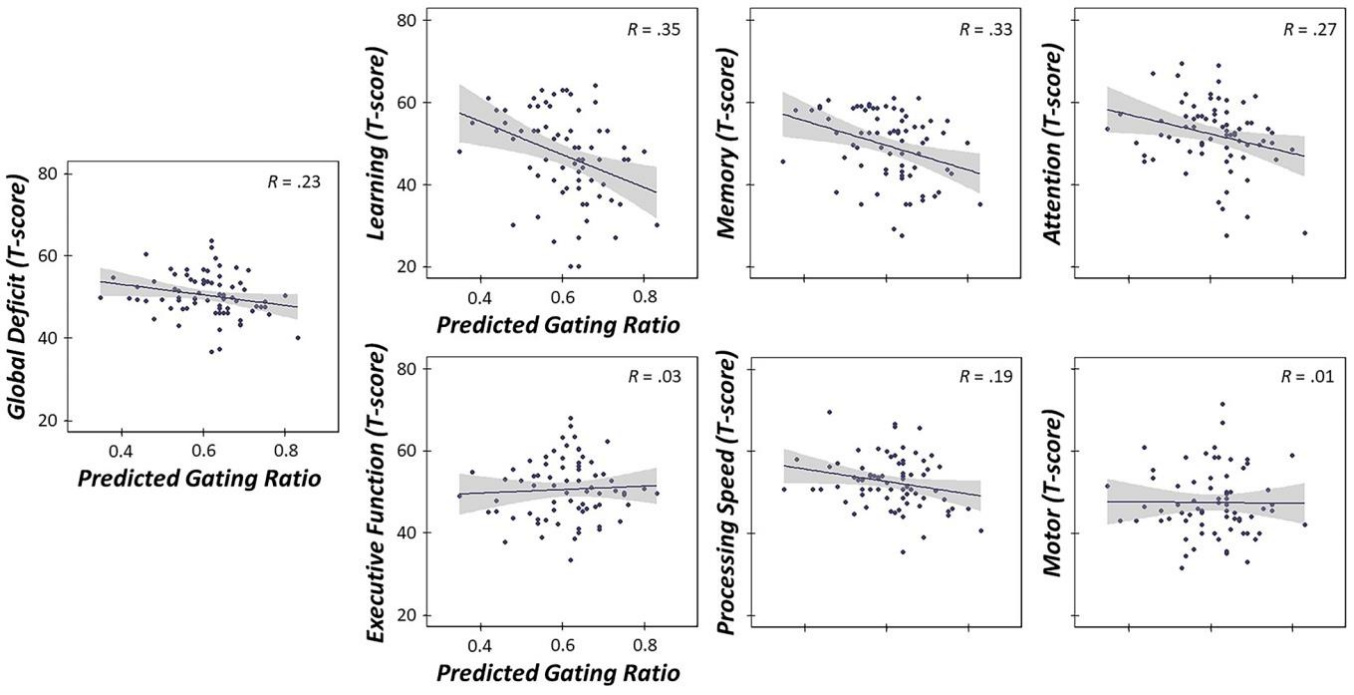
**Stress-induced gating changes predict poorer cognitive outcomes.** Linear regressions of predicted somatosensory gating ratios in the oscillatory domain (accounting for levels of allostatic load, relative age acceleration and their interaction) on six cognitive domain composites (Z-scores) and global cognitive function. Higher gating ratios (i.e., worse suppression of redundant sensory information, accounting for levels of stress) were predictive of poorer global cognition and poorer performance on learning, memory, attention and processing speed domains (*ps* < .028). All axes are fixed for each graph. 95% confidence intervals are displayed in gray for each regression line. *p < .05, **p < .005, ***p < .001.

Finally, given the previous literature suggesting that bottom-up, pre-attentive gating is closely linked with top-down higher order cognitive functions (e.g., attention; (15)), we evaluated whether stress-induced changes in somatosensory gating across the lifespan significantly predicted cognitive function. To assess cognitive function, we used an extensive neuropsychological battery that probed six domains and global cognitive function, including learning, memory, processing speed, executive function, attention, motor function and global cognition. As described in the methods, domain-specific composite scores were computed by averaging the Z-scores of assessments comprising each respective domain. Of note, we used unadjusted Z-scores rather than demographically-normed ones due to the fact that age and years of education were important variables comprising our measures of relative age acceleration and allostatic load, respectively. Next, we calculated the predicted gating ratios accounting for levels of allostatic load, relative age acceleration and their interaction using the regression equation described above. This yielded a predicted value of stress-induced gating, with higher values indicative of worse sensory gating, accounting for levels of life and biological stress. Interestingly, linear regressions of predicted oscillatory gating ratios on each cognitive domain and global cognitive function scores revealed that higher gating ratios (induced by increased levels of stress) were significantly predictive of poorer global cognitive function (i.e., decreased Z-scores averaged across all domains (b = -2.08, *p* = .005, 95% CI [-3.50, -0.66])). In addition, stress-induced declines in sensory gating were also predictive of poorer cognitive function on learning (b = -3.78, *p* = .001, 95% CI [-5.94, -1.62]), memory (b = -3.03, *p* = .003, 95% CI [-5.00, -1.07]), attention (b = -2.17, *p* = .028, 95% CI [-4.10, -0.24]), and processing speed-specific composites (b = -2.07, *p* = .025, 95% CI [-3.86, -0.27]), suggesting that less filtering of redundant sensory information (accounting for elevated levels of stress) led to significantly worse performance on these cognitive domains (Figure 7).

## DISCUSSION

The goal of the current study was to evaluate the independent and cumulative contributions of life and biological stress on the age-related decline in sensory gating. Specifically, we used a paired-pulse electrical stimulation paradigm during MEG to interrogate the gating of oscillatory and evoked neural responses to somatosensory stimulation in a large sample of aging adults (aged 22-72 years old). Using empirically-derived definitions of allostatic load (i.e., life stress) and relative age acceleration (i.e., residuals of DNA methylation predicted biological age on chronological age; biological stress) and structural equation modeling, we observed dissociable mechanisms of action for predicting age-related decline in the gating of gamma oscillations within the primary somatosensory cortex, but not the gating of evoked neural responses. In addition, our study was the first to establish a predictive link between stress-induced changes in bottom-up functional inhibitory processing (i.e., sensory gating) and top-down cognitive dysfunction across the lifespan. The implications for these novel findings are discussed below.

Many recent studies of somatosensory gating have focused on the high-frequency gamma (30-75 Hz) oscillatory dynamics in response to the first and second stimulation in a paired-pulse paradigm, and herein we evaluated the contributions of life and biological stress on the findings noted in these prior studies. Critically, we found that gating of gamma responses in the primary somatosensory cortex was significantly modulated by indices of life, biological and cumulative stress. As expected, greater levels of allostatic load (i.e., life stress), relative age acceleration (i.e., biological stress) and their interaction (i.e., cumulative stress) were significantly predictive of higher gating ratios, indicative of worse suppression of redundant sensory input. Importantly, to facilitate comparison with the broader literature on gating, we also interrogated this relationship with source estimates of evoked neural responses to somatosensory stimulation. Interestingly, neither life, biological nor cumulative measures of stress significantly predicted gating ratios derived from our time-domain analysis, suggesting that evoked and gamma oscillatory activity may be differentially sensitive to stress accumulation across the lifespan. This result is not surprising, as previous investigations by our lab and others have demonstrated dissociable effects on evoked and oscillatory neural responses during somatosensory processing. For example, previous studies have shown that gating ratios derived from oscillatory analysis approaches are more sensitive to subtle external factors, including paradigm shifts (e.g., inter-stimulus interval [39]) and even participant characteristics (e.g., participant age [11]). In a previous study of healthy aging, our lab observed robust increases in the gating ratio based on gamma oscillatory activity, such that older adults exhibited worse suppression of redundant information in the somatosensory cortex [11]. However, gating ratios derived from the time-domain analysis of the same MEG data were not significantly modulated by aging as previously reported by other investigators [10–12].

Indeed, the current study aligns well with prior studies of gamma-mediated mechanisms, and brings attention to the extensive literature implicating GABAergic mechanistic drive in the generation and modulation of gamma activity. In fact, multimodal neuroimaging studies have linked GABA concentration and/or receptor density to gamma response properties (i.e., power and/or frequency) in primary sensory regions in humans, corroborating this well-established link first introduced using cellular electrophysiology [40–49]. For example, a study using GABA magnetic resonance spectroscopy (MRS) and MEG established a link between peak gamma frequency and GABA concentration within the primary motor cortex contralateral to movement, such that increases in gamma frequency were associated with greater GABA concentration [50]. Likewise, Muthukumaraswamy and colleagues were able to demonstrate a similar relationship in the visual system, and related elevations in both peak gamma frequency and GABA concentration to better visual discrimination performance [51, 52]. In regard to healthy aging, previous studies broadly suggest modulation of both gamma and GABA properties as a function of increasing age, such that decreases in peak gamma frequency and GABA concentration are robustly induced by the aging process in primary sensory, as well as frontoparietal networks [50–53]. Taken together, these data suggest that gamma oscillatory activity is critically dependent on GABAergic inhibitory function and, while we did not evaluate GABA directly in this study, we can presume that our stress-induced decline in somatosensory inhibition may be the result of progressive local GABA dysfunction across the lifespan. Importantly, our study was the first to directly link quantitative measures of stress (i.e., allostatic load and relative age acceleration) to gamma oscillatory dynamics during somatosensory inhibition, and future studies explicitly interrogating the role of GABA in this process will be incredibly informative to fully unravel these mechanisms.

A second important finding involved the mechanism of action by which life and biological stress modulate sensory gating. Briefly, our follow up analyses using structural equation modeling revealed that greater levels of allostatic load were predictive of reduced oscillatory power to the first stimulation in the pair, but not the second, while the opposite effect was observed for relative age acceleration. This finding is quite interesting when one considers the factors comprising our measures of life and biological stress. Specifically, our empirically-derived and sample-specific definition of allostatic load suggested that depression symptom severity, body mass index, activities of daily living, and years of education were representative of related life stress factors across our aging population, with higher values indicative of more stress. This composite of factors defining life stress was not surprising, considering the numerous studies linking these factors (i.e., negative outcomes) with the greater physiological dysregulation traditionally defined as impaired allostasis [24, 54–59]. In regard to the brain, each of these factors (e.g., depression, education) alone has been shown to differentially modulate neural responses to novel stimulus presentations. For example, using auditory oddball tasks, investigators have shown a reduction in fronto-central N2 and P3 responses to infrequent or novel auditory stimuli in non-medicated, depressed individuals compared to controls [60, 61]. In addition, a similar reduction in novelty-related responses can be seen for older adults with lower cognitive ability compared to their higher performing counterparts, suggesting a modulation of neural amplitude perhaps based on education, exposure levels, or even IQ [62–65]. While our sensory gating paradigm is quite different from traditional oddball tasks, the presentation of the novel, first stimulation in the pair was significantly reduced with greater levels of allostatic load, suggesting that this stress-induced modulation in somatosensory response strength may be attributable at least in part to the factors comprising life stress (e.g., depression and education) that are known to modulate stimulus novelty in the brain.

In contrast, our results also showed a differential modulation of the gating effect by relative age acceleration/deceleration, such that greater biological age acceleration (compared to chronological age) was associated with less attenuated gamma responses to the second stimulus in the repetitive pair (i.e., the *inhibited* stimulus). Importantly, our study was the first to directly link quantitative measures of age acceleration (DNA methylation age) to gamma oscillatory dynamics in the primary somatosensory cortex and thereby, functional inhibition at the neural population level in humans. Interestingly, this finding may further implicate a role for dysfunctional intracortical inhibition at the cellular level during sensory gating paradigms. Specifically, previous studies have shown that hypermethylated DNA profiles are unique to GABAergic inhibitory interneurons compared to their excitatory or glial cell counterparts in the human prefrontal cortices [66]. In addition, when key epigenetic controllers of DNA methylation are deleted (e.g., methyltransferases), synaptic release of GABA is promoted [67] and the expected age-related loss in primary sensory interneuronal pools are ameliorated and match younger controls in mice [68]. Thus, our results showing that relative age acceleration (derived from DNA methylation predicted age) modulates the gating of redundant somatosensory input through changes in gamma response strength to the second gated stimulus, but not the first, suggests that a GABA-mediated mechanism may underlie the relationship between epigenetic markers of biological stress and functional inhibitory processing of somatosensory input in the brain.

Finally, our most critical finding was likely the stress-induced changes in gating predicting levels of top-down cognitive dysfunction across our aging sample. Specifically, our study was the first to demonstrate that higher predicted gating ratios in the oscillatory domain, accounting for levels of life and biological stress, predicted worse cognitive performance on learning, memory, attention, processing speed, and global functional domains assessed by an extensive neuropsychological battery. While numerous studies of auditory gating have established a relationship between gating ratios and cognitive dysfunction in healthy adults [69–71], only one prior study has evaluated this link in the somatosensory domain. In a previous study of somatosensory gating, Cheng and colleagues established a link between gating ratios during a similar somatosensory paired-pulse paradigm and performance on an attentional task, such that better suppression of somatosensory input (i.e., smaller gating ratios) was associated with increased accuracy on both auditory and somatosensory go/no-go tasks [15]. Our results corroborate the previous literature implicating pre-attentive measures of functional inhibition in attentional control processes, and critically expand our knowledge of these constructs in the somatosensory cortex to learning, memory and processing speed cognitive faculties as well. In addition, our study was the first to evaluate this relationship in the context of healthy aging and, importantly, suggests that stress-induced changes in sensory gating oscillatory dynamics are particularly sensitive to detecting higher-order trajectories of cognitive decline observed across the lifespan.

To close, the aging process is associated with numerous neurobiological alterations, leading to the functional decline observed in later life that is often highly disparate from person to person. The current study evaluated the independent and cumulative contributions of life and biological markers of stress on the age-related decline in functional inhibitory processing using a well-known sensory gating paradigm and high-density MEG. Specifically, we empirically-defined sample-specific indices of life (i.e., allostatic load) and biological (i.e., relative age acceleration: residuals of DNA methylation age on chronological age) stress in a large sample of aging adults (22-72 years old) and observed a robust modulation of somatosensory filtering in the oscillatory domain, but not the time-domain. In addition, we observed a separable mechanism of action by which allostatic load and relative age acceleration modulated the oscillatory gating effect, suggesting that stimulus novelty (i.e., stimulation 1) may be more sensitive to factors comprising life stress, while the inhibited stimulus (i.e., stimulation 2) may be more sensitive to DNA methylation predicted age acceleration. Finally, our study was the first to demonstrate that stress-induced changes in the gating effect were significantly predictive of global and domain-specific cognitive decline across our aging sample. We propose that these trajectories may be the result of GABA-mediated intracortical dysfunction in aging populations [53], given the extensive literature linking GABA interneurons to the modulation of high-frequency pyramidal synchrony (i.e., gamma oscillations) across the cortex [41–45, 47, 49–52, 72]. With the aging population expected to double by 2050 [73], concomitant with increased recognition that not all individuals age equivalently, understanding the factors contributing to age-related variation in functional decline is of utmost importance. Critically, our study supports the notion that markers of stress (including psychosocial, physical and biological) predict age-related decline in pre-attentive functional inhibitory processing (i.e., sensory gating) and further, stress-induced change in sensory gating is particularly sensitive to detecting the cognitive decline observed in aging populations. Together, these findings suggest that the use of sensory gating paradigms in human neurophysiological studies may hold broad, tangible benefits in the long-term, as it could allow for precise detection of healthy and pathological aging trajectories in individual persons.

## MATERIALS AND METHODS

### Participant demographics and neuropsychological assessment

Seventy-four healthy adults (M_age_ = 43.6 years old, range: 22-72 years old, 35 females) were enrolled in this study. Exclusionary criteria included any medical illness affecting CNS function, any neurological or psychiatric disorder, history of head trauma, current pregnancy, current substance use, implanted ferromagnetic objects or extensive dental work, and cognitive impairment. Cognitive impairment was based on a thorough neuropsychological battery that assessed functionality across six domains (i.e., learning, memory, executive function, attention, processing speed, motor function). Participants who scored less than one standard deviation from the mean using demographically-normed scores per test were deemed cognitively impaired and excluded from the study. The battery included the following tests for each domain: learning (Hopkins Verbal Learning Test – Revised (HVLT-R) Learning Trials 1-3 [74]), memory (HVLT-R Delayed Recall and Recognition Discriminability Index [74]), executive function (Comalli Stroop Test Interference Trial [75], semantic verbal fluency [76], phonemic verbal fluency [76], and Trail Making Test Part B [76]), processing speed (Comalli Stroop Test Color Trial [75], Wechsler Adult Intelligence Scale (WAIS-III) Digit Symbol Coding [77], and Trail Making Part A [76]), attention (WAIS-III Symbol Search [77], and Comalli Stroop Word Trial [75]), and motor function (Grooved Pegboard, Dominant and Non-Dominant Hands [76, 78]). Z-scores were computed using raw scores and composite domain-specific scores were calculated by averaging the Z-scores of assessments that comprised each domain respectively (see above). Global cognitive function scores were computed by averaging all domain-specific composite Z-scores. Of note, for a subsample of the participants included in the current study (N = 29), the HVLT-R Learning, Recall and Recognition tests were administered faster than the recommended speed (i.e., approximately 1 word per 1 second rather than 1 word per 2 seconds). However, all statistical analyses including these data accounted for this administration difference, and the strength of these relationships did not differ when accounting for participant exclusions using Fisher Z comparisons (learning: Z = 1.29, *p* = .197; memory: Z = 1.52, *p* = .128). The University of Nebraska Medical Center Institutional Review Board approved the study and all participants provided written informed consent.

### Experimental paradigm

Participants were seated in a nonmagnetic chair with their head positioned within the MEG helmet-shaped sensor array. Electrical stimulation was applied to the right median nerve using external cutaneous stimulators connected to a Digitimer DS7A constant-current stimulator system (Digitimer Limited, Letchworth Garden City, UK). For each participant, we collected at least 80 paired-pulse trials with an inter-stimulation interval of 500 ms and an inter-pair interval that randomly varied between 4500 and 4800 ms. Note that our use of a jittered inter-pair interval of 4500-4800 ms is commonly used to avoid habituation of the neural responses via anticipation of the upcoming paired-pulses [11, 13, 36, 38]. Additionally, our use of 500 ms as the inter-stimulation interval was chosen based on prior studies that have shown that 200-500 ms is the optimal inter-stimulation interval for maximizing gating effects in this paradigm [39]. Each pulse generated a 0.2 ms constant-current square wave that was set to a limit of 10% above the motor threshold required to elicit a subtle twitch of the thumb.

### MEG data acquisition and coregistration with structural MRI

All recordings were performed in a one-layer magnetically-shielded room with active shielding engaged for environmental noise compensation. With an acquisition bandwidth of 0.1-330 Hz, neuromagnetic responses were sampled continuously at 1 kHz using an MEGIN/Elekta MEG system (MEGIN, Helsinki, Finland) with 306 magnetic sensors, including 204 planar gradiometers and 102 magnetometers. Throughout data acquisition, participants were monitored using a real-time audio-video feed from inside the magnetically-shielded room. MEG data from each participant were individually corrected for head motion and subjected to noise reduction using the signal space separation method with a temporal extension [79]. Each participant’s MEG data were coregistered with their structural T1-weighted MRI data prior to imaging analyses using BESA MRI (Version 2.0). Structural MRI data were aligned parallel to the anterior and posterior commissures and transformed into standardized space. After beamformer analysis (see below), each subject’s functional images were transformed into standardized space using the transform that was previously applied to the structural MRI volume and spatially resampled.

### MEG preprocessing and sensor-level statistics

Cardiac and ocular artifacts were removed from the data using signal-space projection (SSP) and the projection operator was accounted for during source reconstruction [80]. Epochs were of 3700 ms duration, with 0 ms defined as the onset of the first stimulation and the baseline being the -700 to -300 ms window. Of note, we shifted our baseline away from the period immediately preceding stimulus onset to avoid potential contamination by any anticipatory responses, although there was no evidence of such anticipatory responses in our final analyses. Epochs containing artifacts were rejected based on a fixed threshold method, supplemented with visual inspection. On average, 73 trials per participant were used for further analysis.

Artifact-free epochs were further processed using two parallel pipelines. For the oscillatory analysis, epochs were transformed into the time-frequency domain using complex demodulation [81], and the resulting spectral power estimations per sensor were averaged over trials to generate time-frequency plots of mean spectral density. The sensor-level data per time-frequency bin were normalized using the mean power per frequency during the -700 to -300 ms baseline period. The specific time-frequency windows used for imaging were determined through a two-stage, data-driven approach involving statistical analysis of the sensor-level spectrograms across all participants and trials. First, paired-sample t-tests against baseline were conducted on each data point, with the output spectrogram of t-values initially thresholded at *p* < .05 to define time-frequency bins containing potentially significant oscillatory deviations. To reduce the risk of false positive results due to multiple comparisons, the time-frequency bins that survived that initial threshold were temporally and/or spectrally clustered with neighboring bins that were also significant, and a cluster value was derived by summing all of the t-values of all data points in the cluster. Nonparametric permutation testing (10,000 permutations) was then used to derive a distribution of cluster values and the significance level of the observed clusters were tested directly using this distribution. Based on this analysis, the time-frequency periods that contained significant oscillatory events across all participants were subjected to beamforming analyses. Of note, in the case of the broadband gamma oscillations, we focused on a window surrounding the peak of the response (i.e., greatest amplitude change from baseline) in order to optimize the signal to noise ratio. Note that the significant time-frequency extent of the gamma response extended beyond this 75 Hz band, especially following the first stimulation.

For the time domain (i.e., evoked) analyses, all artifact-free epochs per participant were averaged with respect to the onset of the first stimulation for each sensor in the array, and the resulting mean time series per sensor and participant were examined statistically to determine the specific time windows used for subsequent source analyses. Like the oscillatory analyses, we used a two-stage approach that included paired-sample t-test against baseline, followed up with cluster-based permutation testing to control for multiple comparisons (initial threshold: *p* < .05, permutations: 10,000). The phase-locked, time-domain period that significantly differed from baseline were used to guide subsequent time-domain source level analysis. Further details of this method and our processing pipeline can be found in recent papers [6, 11, 36, 38, 39].

### MEG source imaging

Cortical oscillatory networks were imaged through the dynamic imaging of coherent sources (DICS) beamformer [82], which uses the cross-spectral density matrices to calculate source power for the entire brain volume. These images are typically referred to as pseudo-t maps, with units (pseudo-t) that reflect noise-normalized power differences (i.e., active vs. passive) per voxel. Following convention, we computed noise-normalized, source power per voxel in each participant using baseline periods of equal duration and bandwidth [83]. MEG preprocessing and imaging used the Brain Electrical Source Analysis (Version 6.1; BESA) software. Further details of our analysis pipeline can be found in Spooner et al., (2020) [39].

Normalized source power was computed over the entire brain volume per participant at 4.0 × 4.0 × 4.0 mm resolution for the time-frequency periods identified through the sensor level analyses. Prior to statistical analysis, each participant’s MEG data, which were coregistered to native space structural MRI prior to beamforming, were transformed into standardized space using the transform previously applied to the structural MRI volume and spatially resampled. The resulting 3D maps of brain activity were averaged across all participants and both stimulations to assess the neuroanatomical basis of the significant oscillatory responses identified through the sensor-level analysis, and to allow identification of the peak voxels per oscillatory response.

Voxel time series data (i.e., “virtual sensors”) were extracted from each participant’s data individually using the peak voxel from the grand-averaged beamformer images. To compute the virtual sensors, we applied the sensor weighting matrix derived through the forward computation to the preprocessed signal vector, which yielded a time series for the specific coordinate in source space. Note that virtual sensor extraction was done per participant, once the coordinates of interest were known. Once the virtual sensor time series were extracted, we computed the envelope of the spectral power within the frequency range used in the beamforming analysis. From this time series, we computed the relative (i.e., baseline-corrected) response time series of each participant to quantify indices of somatosensory processing, including the gating ratio (response power to stim 2/response power to stim1) and source power in response to both electrical stimulations.

To enhance comparability with previous work, source images of the time-domain averaged responses were computed using standardized low-resolution brain electromagnetic tomography (sLORETA; regularization: Tikhonov 0.01%) [84]. The resulting whole-brain maps were 4-dimensional estimates of current density per voxel, per time sample across the experimental epoch. These data were normalized to the sum of the noise covariance and theoretical signal covariance, and thus the units are arbitrary. Using the temporal clusters identified in the time-domain sensor-level analysis, these maps were averaged over time following each somatosensory stimulation (e.g., 25-125 ms and 525-625 ms). The resulting maps were then grand-averaged across the two stimulations to determine the peak voxel of the time-domain neural response to the stimuli across participants. From this peak, voxel time series were extracted in sLORETA units to quantify measures of somatosensory processing (i.e., gating ratio, source power).

### Blood-based markers of age acceleration

To evaluate levels of biological stress on age-related changes in sensory gating, whole blood samples were collected from each participant using BD Vacutainer EDTA tubes to evaluate methylation metrics based on the Hannum, Horvath and consensus models of predicted biological age. DNA methylation analyses were conducted on the entire data set from a large study of aging adults (N > 180) reported in previous publications [11, 13, 38, 85–87] and closely aligned with epigenetic age estimations established in previous work [35].

Specifically, DNA was purified from whole blood using DNeasy blood tissue extraction kits (QIAGEN). Methylation analysis was performed using Infinium HumanMethylation450 BeadChip Kits (Illumina). Following hybridization, BeadChips were scanned using the Illumina HiScan System. All data were processed through the Minfi R processing pipeline [88]. Methylome data were downloaded from Hannum [26] and EPIC [89] (GEO: GSE40279 and GSE51032), and these data were processed together along with methylation data generated from the larger study mentioned above. Beta values were extracted and quantile normalized using Minfi; cell counts were estimated using estimateCellComposition and resulting normalized beta values were adjusted for cell types [35, 90]. All data was then normalized using a modified BMIQ procedure provided by Horvath [25]. The gold standard was set to the median beta observed in the Hannum study [26].

For the current study, the “consensus model” of predicted biological age (i.e., both Hannum and Horvath predictions) was used, as this has been shown to outperform either prediction model in isolation [35]. Importantly, our measure of relative age acceleration (i.e., acceleration or deceleration in biological age relative to chronological age) was computed using the residuals from a regression of the consensus model of predicted biological age on chronological age for the 74 participants eligible for the current study.

### Definition of allostatic load

To index relevant markers of allostatic load (i.e., life stress) in the current sample, we conducted an exploratory factor analysis (EFA) to define a latent variable of allostatic load using a compilation of metrics that are known to contribute to overall life stress and health. We began with a set list of seven measurements and progressively removed individual variables based on poor loadings (λ > .20), and overall model fit. Criteria for good model fit included a non-statistically significant chi square, a root mean squared error of approximation (RMSEA) < .06, a comparative fit index (CFI) > .95, and a standardized root mean squared residual (SRMR) < .08 based on standards in the literature [91]. The best-fitting model was used to define a latent variable for which an allostatic load score was extracted per participant. Modeling was completed using Mplus (Version 8.1).

We began by fitting a latent variable defined by alcohol use (Alcohol Use Disorders Identification Test—Composition Score (AUDIT-C)), relative age acceleration, depression (Beck Depression Index total score), number of self-reported declines in activities of daily living (ADL), body mass index (BMI), household income, and years of education. Briefly, alcohol use was defined using the AUDIT-C [92], which contains items that reflect the amount and/or frequency of current and past alcohol use during the past year. Thus, participants with higher scores are defined as heavy alcohol users with scores of four or more for men and three or more for women being considered clinically-relevant heavy alcohol use. In regard to depression symptom severity, we used the Beck Depression Inventory-II (BDI-II [93]), which requires participants to answer questions regarding the presence of depressive symptoms within a two-week period, with higher scores indicative of greater symptom severity. Regarding ADL, these scores were determined based on self-report declines in the participant’s ability to carry out daily tasks (e.g., dressing, eating, personal hygiene, etc.). Similar to our other measures, higher ADL scores reflected greater perceived decline in performance of daily tasks. Income and education were reverse-coded such that increasing values reflected lower education and income, and thus were indicative of greater stress risk. Importantly, all measures included in the EFA were treated as continuous variables of potential life stress. The resultant latent variable yielded allostatic load scores in which higher values indicated more allostatic load (i.e., greater life stress). The initial EFA based on all seven life stress factors indicated a single-factor solution with excellent fit (χ^2^(14) = 14.92, *p* = .38; RMSEA = .03, 90% CI [.00, .12]; CFI = .96; SRMR = .06). However, AUDIT-C, relative age acceleration, and income all loaded poorly onto the factor (λ’s = -.009 to .032). Excluding these three variables yielded a superior EFA solution, still yielding a single factor (λ’s = .22 to .80) with excellent overall model fit (χ^2^(2) = 2.03, *p* = .36; RMSEA = .01, 90% CI [.00, .23]; CFI = .99; SRMR = .04). Thus, our empirically-defined sample-specific quantification of allostatic load was comprised of depression symptom severity, total years of education, perceived declines in ADL, and BMI, with higher values indicative of greater current life stress (Figure 1). An allostatic load score was extracted per participant using the structure of this final EFA solution.

### Statistical analyses

To evaluate the predictive capacity of life and biological markers of stress on age-related declines in somatosensory gating, we conducted a series of regressions with allostatic load (i.e., life stress), relative age acceleration (i.e., biological stress) and their interaction (i.e., cumulative stress; allostatic load score x relative age advancement) as predictors of the gating ratio derived from oscillatory and time-domain source imaging techniques, separately (Figure 1). In addition, we conducted follow-up analyses to examine the mechanism of action by which life and biological stress modulated the age-related decline in sensory gating. Specifically, we examined whether life and biological stress differentially predicted the neural response power to the first and second stimulation in our paired-pulse paradigm using structural equation modeling in Mplus (Version 8.1). Finally, we aimed to examine whether stress-induced changes to bottom-up sensory gating were predictive of higher order cognitive function assessed outside of the scanner using an extensive neuropsychological battery. Essentially, we computed a predicted gating ratio per participant accounting for levels of allostatic load, relative age acceleration and their interaction (i.e., using the regression equation described above), with higher values indicative of worse suppression of redundant sensory input. Next, we conducted a series of linear regressions of predicted gating ratios on six cognitive domains and global cognitive function (Z-scores). Of note, we used unadjusted Z-scores to compute domain-specific composites rather than demographically-normed ones due to the fact that age and years of education were important variables comprising our measures of relative age acceleration and allostatic load, respectively.
